# Quantitative model for rationalizing solvent effect in noncovalent CH–Aryl interactions†
†Electronic supplementary information (ESI) available: Correlation plots; proton and carbon NMR spectra of the molecular balances, and the *xyz* coordinate of the optimized geometries. See DOI: 10.1039/c5sc03550c


**DOI:** 10.1039/c5sc03550c

**Published:** 2015-11-17

**Authors:** Bright U. Emenike, Sara N. Bey, Brianna C. Bigelow, Srinivas V. S. Chakravartula

**Affiliations:** a Department of Chemistry & Physics , State University of New York , 223 Store Hill Road, Old Westbury , NY 11568 , USA . Email: emenikeb@oldwestbury.edu; b Department of Chemistry & Biochemistry , Hunter College Graduation Center , City University of New York , 695 Park Avenue New York , NY 10065 , USA

## Abstract

The strength of CH–aryl interactions (Δ*G*) in 14 solvents was determined *via* the conformational analysis of a molecular torsion balance. The molecular balance adopted folded and unfolded conformers in which the ratio of the conformers in solution provided a quantitative measure of Δ*G* as a function of solvation. While a single empirical solvent parameter based on solvent polarity failed to explain solvent effect in the molecular balance, it is shown that these Δ*G* values can be correlated through a multiparameter linear solvation energy relationship (LSER) using the equation introduced by Kamlet and Taft. The resulting LSER equation [Δ*G* = –0.24 + 0.23*α* – 0.68*β* – 0.1π* + 0.09*δ*]—expresses Δ*G* as a function of Kamlet–Taft solvent parameters—revealed that specific solvent effects (*α* and *β*) are mainly responsible for “tipping” the molecular balance in favour of one conformer over the other, where *α* represents a solvents' hydrogen-bond acidity and *β* represents a solvents' hydrogen-bond basicity. Furthermore, using extrapolated data (*α* and *β*) and the known π* value for the gas phase, the LSER equation predicted Δ*G* in the gas phase to be –0.31 kcal mol^–1^, which agrees with –0.35 kcal mol^–1^ estimated from DFT-D calculations.

## Abstract 2

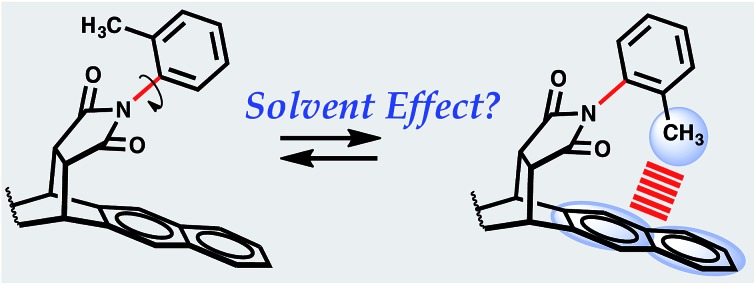
Establishing a linear relationship between CH–aryl interaction energies and the properties of the solvating media.

## Introduction

Noncovalent CH–aryl interactions are of fundamental importance for molecular recognition in a vast number of chemical and biological processes.^1^–^3^ Although studies have revealed the physical origins of CH–aryl interactions,^4^,^5^ the effects of solvation have not been thoroughly examined, at least not from a quantitative perspective. Various theoretical calculations by density functional theory (DFT) estimate the interaction energy between a methyl group and the face of a benzene ring to range between –1.0 to –1.5 kcal mol^–1^.^5^,^6^ However, the energetics of CH–aryl interactions observed in solution are comparatively much lower (less than –0.4 kcal mol^–1^ in chloroform).^7^–^9^ The disparity between experimental results and DFT calculations hinges on the effect from the explicit solvent interaction, which alters the net forces governing a CH–aryl interaction in solution. For example, it has been shown that a significant portion of CH–aryl interaction occurring in the gas phase originates from electrostatic and London dispersion forces.^5^,^6^,^10^,^11^ On the other hand, however, the net effect of London dispersion forces for interactions taking place in solution has been reported to be negligible.^12^,^13^ Although theoretical methods offer a convenient way of studying CH–aryl interactions, they seldom capture the complicated effects of solvation phenomenon, which is problematic particularly because the explicit interactions between solute and solvent molecules are an integral part of solvation.^14^

The surprising lack of research interest in a quantitative model of solvent effects in CH–aryl interactions is not because solvent effects are unimportant, but, rather, due to the following reasons: (1) there is a relative lack of data on the strengths of CH–aryl interactions in various solvents, and (2) there have not been widely accepted models to quantitatively rationalize solvent effects in CH–aryl interactions. These reasons are further complicated by the energetically weak nature of CH–aryl interactions, which makes experimental measurements of CH–aryl interactions in solution a nontrivial task. Therefore, the first goal of this study is to employ a series of molecular torsion balances—capable of measuring small interaction energies—as model systems to experimentally quantify the strength of CH–aryl interactions as a function of solvation. The second objective is to explore the possibility of constructing a quantitative model—using a *linear solvation energy relationship*—to describe the energetics of CH–aryl interactions as a function of the properties of the solvating media.

## Results and discussion

### Model system

**Fig. 1:**
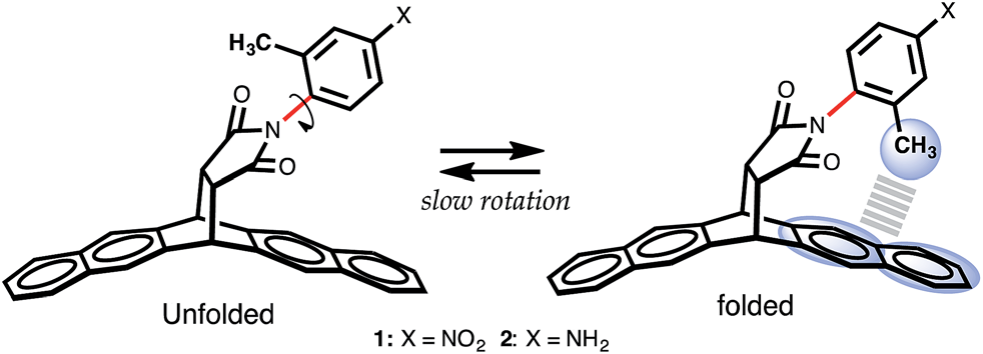
Scheme showing folded and unfolded conformational states of molecular torsional balance **1** and **2**. For structural details, see ref. 16 for related single crystal structures.^16^

The model system chosen to accomplish these objectives is based on the architectural carbon framework of bicyclic *N*-arylimides, Fig. 1. Previous studies by Harano *et al.*,^15^ Shimizu *et al.*,^9^,^17^,^18^ Verma *et al.*,^19^ Grossmann *et al.*,^20^ and Yamada *et al.*^21^ have demonstrated that *N*-arylimide molecular balances exhibit slow rotation about the C_aryl_–N_imide_ bond at room temperature (Δ*G*^‡^ ∼ 20 kcal mol^–1^).^9^,^22^ The slowly rotating C_aryl_–N_imide_ bond (indicated in red in Fig. 1) creates a two-state dynamic system that gives rise to two conformational states: “folded” and “unfolded”. In the folded state, the *ortho* methyl is positioned over the π electron cloud of the naphthalenyl ring in a fashion that makes it plausible for a CH–aryl interaction to take place. The population of each conformational state is determined by integrating the two distinctive peaks (using proton NMR spectroscopy) that correspond to the *ortho* methyl proton or that of the *ortho* phenyl proton in the folded and unfolded conformers. Subsequently, the free energy of the CH–aryl interaction (Δ*G*_exp_ in kcal mol^–1^) is calculated from the ratio of the population of the conformational states following the Gibbs relation, eqn (1). In the absence of the CH–aryl interaction, the molecular balance is expected to have no conformational preferences, *i.e.*, the percentage of folded conformer (*F*_c_) = 50%. Consequently, *F*_c_ > 50% is indicative of an attractive CH–aryl interaction and *F*_c_ < 50% suggests a repulsive CH–aryl interaction.

1 Δ*G* = –*RT* ln *K* = –*RT* ln[folded]/[unfolded]

**Scheme 1:**
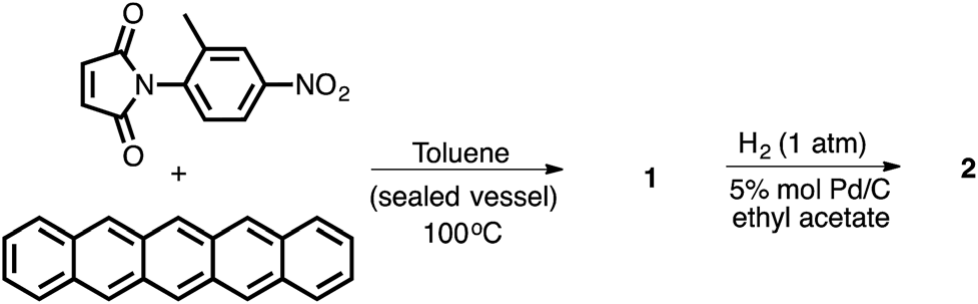
Synthetic route to molecular torsion balances **1** and **2**.

One of the appealing features of the *N*-arylimide based molecular balances is that they can be readily synthesized,^16^ as demonstrated in Scheme 1. The convergent Diels–Alder reaction of the imide and pentacene furnished molecular balance **1** in a quantitative yield. Subsequent reductive hydrogenation of **1** using Pd/C under 1 atm of H_2_ gas produced balance **2** in a similarly high yield.

In order to test the dependence of the conformational preferences on the CH–aryl interaction, the electron density of the π system forming a contact with the CH donor was reduced. The balance variants in which the methyl group could form contacts with a naphthyl, phenyl and ethenyl groups were synthesized (compounds **1** to **4**, respectively).

Because the polarity of the *ortho* methyl protons is expected to remain unperturbed by these modifications, only the electron density of the π face should be affected. Gratifyingly, the results from the proton NMR analysis—taken in DMSO-*d*_6_ solution—indicated a reduction in the strength of the CH–aryl interaction as the percentage of folded conformer (*F*_c_) value dropped from 82% (for **1**) to 68% (for **3**) and to 58% (CH–ethenyl interaction for **4**). This trend can be explained by the observation that the systematic depletion of π electron density led to a gradual reduction in the strength of CH–aryl interaction. Concomitantly, the magnitude of the expected “shielding” effect caused by the aromatic ring current—measured as the difference in the chemical shifts (*δ*) of the methyl protons in the two conformers—is largest for balance **1** (Δ*δ* = 1.65 ppm), followed by balance **3** (Δ*δ* = 1.08 ppm), and smallest for balance **4** (Δ*δ* = 0.08 ppm).

**Fig. 2:**
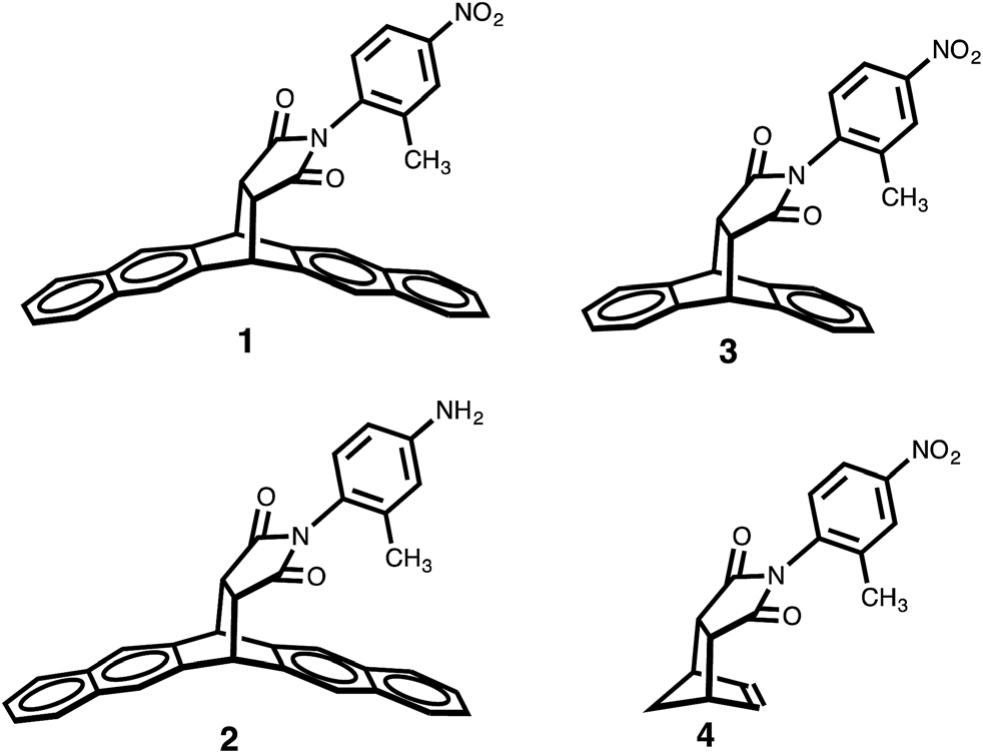
Structures of molecular balances **1–4** used in the solution studies of CH–aryl interactions.

Following the same line of thought, the effect of CH acidity (on the conformational preferences of balance **1**) was probed by tuning the polarity of the *ortho* methyl with substituents, and, with the π electron density of the naphthalene ring remaining unperturbed. The nitro group placed at the rotating phenyl group (in balance **1**) is expected to polarize the CH group through a positive inductive effect, which will likely enhance the population of the folded conformer should the origin of the conformational preference rely on the strength of the CH–aryl interaction. Relative to balance **2**—which possess an electron-donating amino substituent—the proton NMR analysis (in DMSO-*d*_6_ solution) indicated that the CH–aryl interaction in **1** was indeed stronger than the CH–aryl interaction in **2** because the populations of folded conformer (*F*_c_) were 82% and 77%, respectively. Although the substituent effect was only marginal, the fact that the *F*_c_ values in both balances are well above the threshold value of 50% provides indications that “strong” interaction, perhaps a CH–aryl interaction, stabilizes the folded state. Furthermore, DFT optimization at B3LYP/6-31G+(d) level shows that both conformers have approximately the same dipole moments (*e.g.*, 7.7 D for folded conformer, and 7.4 D for unfolded conformer), which alleviates potential concerns that solvent effects might simply be a reflection of the difference in the polarities of the conformers. Altogether, these results emphasize the importance of CH–aryl interactions on the conformational preference of the molecular balances (Fig. 2).

### CH–aryl interactions in solution

Having shown that balance **1** is a viable probe for measuring a CH–aryl interaction, we proceeded to obtain *F*_c_ values in 14 different solvents (Table 1). The results show that CH–aryl interactions are favourable in polar solvents and slightly unfavourable in nonpolar solvents. The experimental interaction energies span a range of –0.22 kcal mol^–1^ for the weakest CH–aryl interaction in cyclohexane to –0.90 kcal mol^–1^ for the strongest CH–aryl interaction in dimethylsulfoxide (DMSO). Qualitatively, these results are consistent with recent ideas that suggest solvophobic effects are responsible for interactions between nonpolar functional groups.^23^,^24^

**Table 1:** Predicted and measured folding energies of balance **1**, and the Kamlet–Taft solvent parameters

#	Solvent^*b*^	*α* ^*e*^	*β* ^*e*^	π*^e^	*F*_c_ ^*c*^	Δ*G*_exp_^*a*^	Δ*G*_pred_^*d*^
1	Cyclohexane	0.00	0.00	0.00	59	–0.22	–0.24
2	Chloroform	0.20	0.10	0.58	61	–0.26	–0.27
3	CD_2_Cl_2_	0.13	0.10	0.82	61	–0.26	–0.31
4	CCl_4_	0.00	0.10	0.28	63	–0.31	–0.29
5	Benzene	0.00	0.10	0.59	64	–0.34	–0.28
6	Pyridine	0.00	0.64	0.87	74	–0.62	–0.67
7	DMSO	0.00	0.76	1.00	82	–0.90	–0.85
8	*p*-Dioxane	0.00	0.37	0.55	71	–0.53	–0.54
9	Methanol	0.98	0.66	0.60	70	–0.50	–0.52
10	Acetic acid	1.12	0.45	0.64	65	–0.37	–0.35
11	Acetone	0.08	0.43	0.71	73	–0.59	–0.58
12	Acetonitrile	0.19	0.40	0.75	73	–0.59	–0.54
13	THF	0.00	0.55	0.58	76	–0.68	–0.67
14	CD_3_NO_2_	0.22	0.06	0.85	64	–0.34	–0.31

^*a*^Values in kcal mol^–1^ with associated errors of 0.03 kcal mol^–1^.^27^

^*b*^Deuterated solvents were used unless stated otherwise.

^*c*^Values are in %.

^*d*^Values are in kcal mol^–1^, see ESI for associated errors.

^*e*^Values for Kamlet–Taft parameters are obtained from literature.^28^

**Fig. 3:**
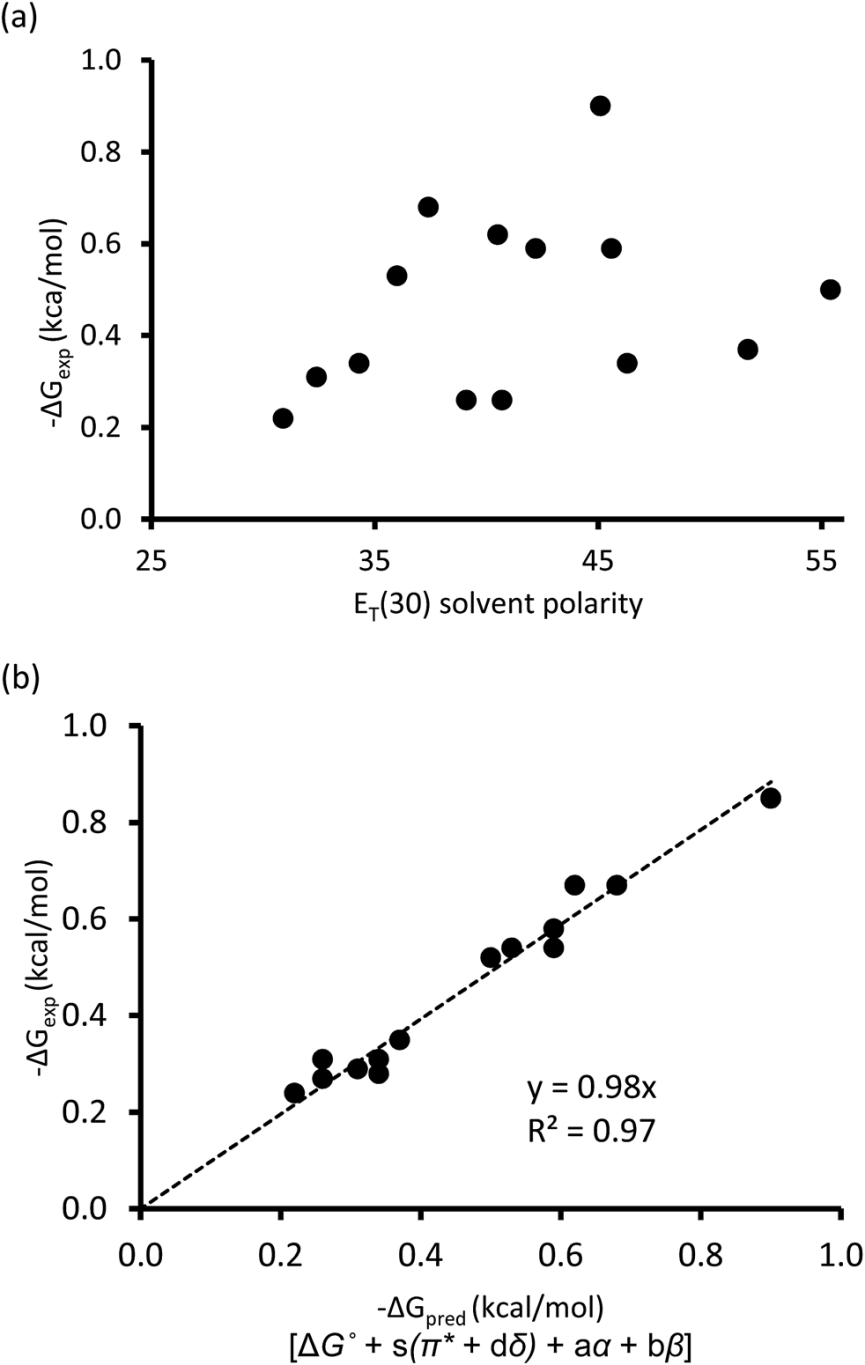
(a) Correlation plot of experimental Δ*G*_exp_ with solvent *E*_T_(30) polarity scale. (b) Linear solvation energy relationship constructed with Kamlet–Taft solvent parameters.

One of the traditional methods to attempt to rationalize solvent effects in molecular recognition studies is to simply relate the interaction energies as a function of solvent polarity.^25^ However, this approach is not straightforward because the term “solvent polarity” is a loosely defined concept, which can be arbitrarily interpreted as the permanent dipole moment (*μ*) of the solvating molecule, the dielectric constant (*ε*), or the solvent polarity parameter *E*_T_(30) of the solvating media.^14^,^26^ Nonetheless, the linear correlation of these solvent polarity parameters only led to weak correlations (Fig. 3a, also see ESI†). Furthermore, the possible correlation between the interaction energies and the cohesive energy density (ced) of the solvents was also explored. Cockroft *et al.* recently introduced the ced solvation model as a single universal descriptor for rationalizing solvophobic effects in nonpolar interactions.^23^ Unfortunately, the ced model only resulted in a poor correlation (*R*^2^ = 0.19, see ESI†) when applied to balance **1**. The poor correlations between Δ*G*_exp_ and the *E*_T_(30) solvent polarity parameter and that of the ced model suggest that single and implicit solvent parameters do not produce an adequate model to quantitatively account for the observed solvent effect in the balance system. Alternatively, an approach that takes into account not only the nonspecific properties of solvation, but also specific aspects of solvation, appears to be a more *complete* treatment of solvation. Fortunately, the multiparameter methods—also known as *linear solvation energy relationship* (LSER)—developed by Kamlet and Taft,^29^,^30^ quantitatively partitions multiple solvent effects occurring differently or simultaneously into their respective contributors based on the solvents' electron pair-sharing (specific) parameters and polarity (nonspecific) parameters. Eqn (2) is the generalized form of the Kamlet–Taft LSER.

2 XYZ = XYZ° + *aα* + *bβ* + *s*(π* + *dδ*)

In eqn (2), XYZ is a solvent-dependent property of interest that usually includes rate constant, equilibrium constants or, in the context of the present study, Δ*G*_exp_. XYZ° is a constant derived from the multiple linear regression of eqn (2), and it equals XYZ in cyclohexane (as the reference solvent). The ability of solvent molecules to accept or donate a lone pairs of electrons towards XYZ process is represented by *α* and *β* terms, respectively, which also denotes hydrogen-bond acidity and hydrogen-bond basicity, respectively. The effect of dipole–dipole interaction on the XYZ property is denoted by the so-called dipolarity–polarizability term, *s*(π* + *dδ*), where π* is generally proportional to the molecular dipole moment of the solvent molecule with single dominant dipole moments, and *δ* is a polarizability correction factor (1.0 for aromatic solvents; 0.5 for polychlorinated solvents; and 0.0 for all other solvents). The magnitudes and the signs of the coefficients (*a*, *b*, and *s*) resulting from the multiple linear regression of eqn (2) provide a measure of the relative susceptibility of the XYZ physicochemical process to the indicated solvent property scales. The Kamlet–Taft solvation model has been successfully used to rationalize solvent effects in a variety of processes, including but not limited to, solubility partition coefficients,^31^,^32^ reaction rates,^33^ and conformational preferences.^30^,^34^ However, to the best of our knowledge, it has yet to be adopted as a quantitative model for rationalizing solvent effect in weak noncovalent interactions such as that of a CH–aryl interaction.

Using entries 1–10 (Table 1) as a *training* set, the multiple regression analysis of Δ*G*_exp_ (dependent variable) against Kamlet–Taft parameters (independent variables), produced a linear relationship (eqn (3)). At 95% confidence level, and with correlation coefficient (*R*^2^) of 0.97 and standard deviation of 0.05 kcal mol^–1^, the Kamlet–Taft solvation model effectively captured the solvent effect in our balance system. This correlation provided a good fit to all data on a single line without any major outliers (Fig. 3b). From the point of predicting solvent effects in a CH–aryl interaction, this result represents a significant advancement over previous models. The validity of eqn (3) was corroborated with four additional solvents (Table 1, entry 11–14), in which the predicted free energy change (Δ*G*_pred_) closely matched experimental Δ*G*_exp_ values (within error). Interestingly, despite the incorporation of a nitro group in balance **1**, which was expected to “polarize” the *ortho* methyl and cause the CH–aryl interaction to have a polar/π-like character, the observed solvent effect was nevertheless reminiscent of interactions between two nonpolar fragments.

In the context of solvophobic effect, it appears that the solvation of the “exposed” *ortho* methyl and naphthalenyl surfaces by nonpolar solvents stabilized the unfolded state while desolvation, facilitated by polar solvents, drove the molecule balance towards the folded state.

3 Δ*G*_exp_ = –0.24 + 0.23*α* – 0.68*β* – 0.1π* + 0.09*δ*

In spite of the weak dependence of Δ*G*_exp_ on single solvent parameter (as shown in Fig. 3a), the specific hydrogen bond parameters, *α* and *β* terms, were found to be dominant contributors because the combined coefficients (*a* and *b*) were significantly larger than the values of the *s* coefficient. In fact, Δ*G*_exp_ showed a strong linear relationship with only *α* and *β* terms (*R*^2^ = 0.92, see ESI Fig. S2†). This result is important because it demonstrates that even for interactions between nonpolar functional groups, it is apparently more accurate to treat solvation as a specific interaction between solute and solvent molecules rather than as a non-structured continuum. This result also demonstrates that solvents' hydrogen bond parameters (*α* and *β*) are apparently more important than dispersion forces for interactions taking place in solution. The contributions of dispersion forces are estimated by the small coefficient of the π* term.

In order to further corroborate the dependence of Δ*G*_exp_ on the solvents' hydrogen bond properties, we also tested the Hunter solvation model,^35^–^37^ which is based on a set of hydrogen bond parameters (*α*_s_ and *β*_s_) that are derived differently from those of the Kamlet–Taft parameters. Interestingly, the Hunter model showed a correlation of *R*^2^ = 0.93 between Δ*G*_exp_ and the *α*_s_ and *β*_s_ values (see ESI†), which was almost as good as the Kamlet–Taft correlation.

Although, both Kamlet–Taft *α* and *β* terms are dominant contributors towards the observed solvent effect, the opposite signs of the coefficients (*i.e.*, + 0.23 for *a* and –0.68 for *b*) suggest that solvents with high *β* values stabilize the folded state while solvents with high *α* values tilt the conformational preference towards the unfolded state. This opposing trend can be rationalized on the basis of explicit solvent–solute interactions. Solvents with high *β* values (*i.e.*, high electron-pair donors) are likely to avoid the naphthalene's π cloud because of the energetically unfavourable lone pair-π interaction, which in turn, favours the folded state. On the other hand, solvents with high *α* values (*i.e.*, high electron-pair acceptors) will engage in a favourable formal hydrogen bond with the naphthalene π electrons, which will bias the conformational preferences in favour of the unfolded state. An alternative yet complimentary rationale is based on the difference in the polarities of the *ortho* aryl CH and the *ortho* methyl group. The NO_2_ substituent more strongly polarizes the aromatic CH groups than the methyl group;^38^ consequently, in solvents with high *β* constants, it is energetically preferable for the *ortho* aromatic CH group to be exposed to the solvent (*i.e.*, the folded conformation is stabilized).

### CH–aryl interactions in the gas phase

Because eqn (3) describes Δ*G* as a function of Kamlet–Taft parameters, it follows that if the *α*, *β*, and π* values are known for the gas phase or conditions that mimic the gas phase, then Δ*G*_pred_ should, in principle, be equal to the interaction energy expected in the gas phase. However, because Kamlet–Taft parameters were developed with cyclohexane as the reference solvent, Δ*G* predicted with *α*, *β*, and π* values all equal to zero does not represent an absence of solvent effect; rather these zero values simply represent interaction energies equivalent to those existing in a cyclohexane solution. Therefore, the questions of interest at this point are: what are the values for *α*, *β*, and π* in the gas phase, and will such quantities accurately predict the energy of the CH–aryl interaction in the gas phase?

**Fig. 4:**
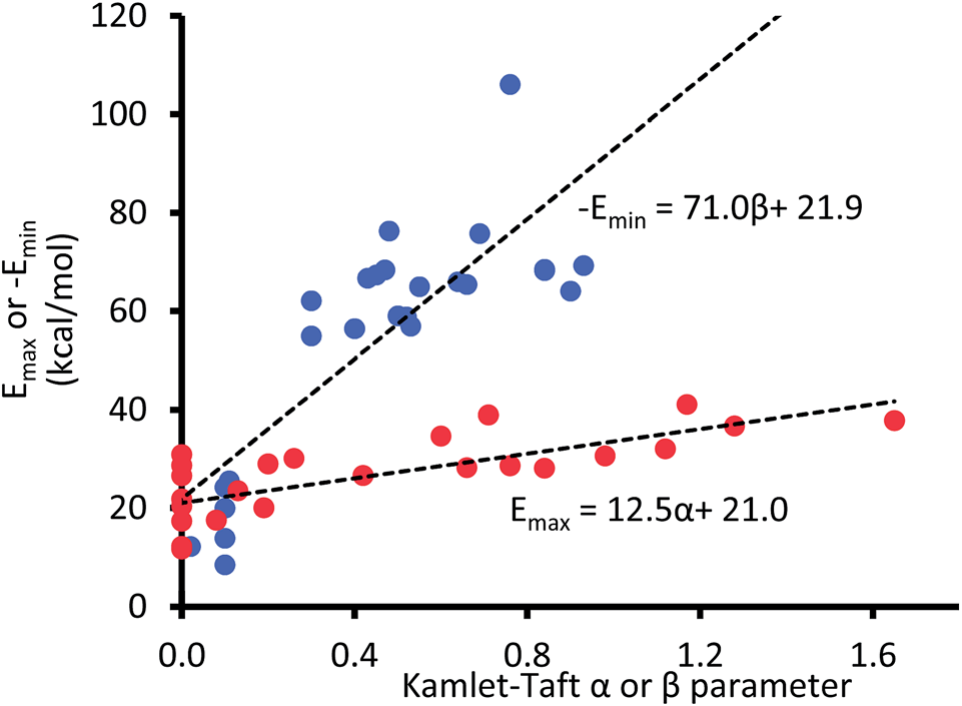
The maxima (*E*_max_) and minima (*E*_min_) in the AM1 molecular electrostatic potential surfaces of 24 solvent molecules.

Unlike the π* value of –1.1 units reported in the gas phase,^39^ to the best of our knowledge, *α* and *β* values in the gas phase have not yet been reported in the literature. However, few reports have shown that energy maxima (*E*_max_) and energy minima (*E*_min_) in the electrostatic potential (ESP) surface of the solvating molecule are directly proportional to solvents' hydrogen bond donor and acceptor parameters, respectively.^40^–^42^ Therefore, the unknown *α* and *β* values for a given solution can be estimated from the ESP surface of the solvating molecule. Note that the energy maxima (*E*_max_) and energy minima (*E*_min_) of most solvents are likely to be a non-zero value (*i.e.*, *E*_max_ > 0 kcal mol^–1^ and *E*_min_ < 0 kcal mol^–1^), and we are assuming that an ideal solvent that closely mimics the gas phase condition or vacuum is one in which the ESP surface is “non-interactive”, *i.e.*, *E*_max_ and *E*_min_ are both equal 0 kcal mol^–1^. In other words, the intercepts on the horizontal axes in the plots of *α* and *β* as a function of electrostatic ESP energies (shown in Fig. 4) should correspond to *α* and *β* values in the gas phase.

Even at the low level AM1 theory, the calculated *E*_max_ and *E*_min_ in the ESP surfaces correlate well with Kamlet–Taft *α* and *β* values, respectively, which is consistent with the findings by Hunter *et al.*^40^

**Fig. 5:**
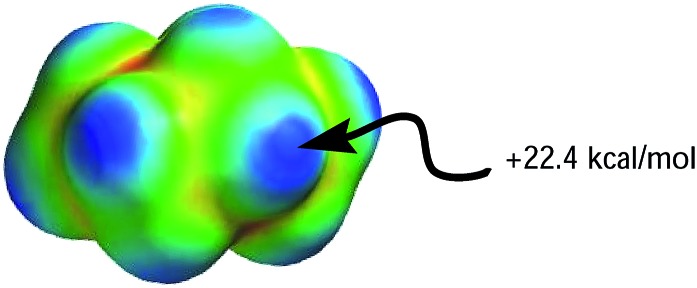
Molecular electrostatic potential surface plotted on the van der Waals' surface of cyclohexane calculated using AM1 level of theory.

Although both plots intersect the vertical axis at ∼22 kcal mol^–1^, the *β* plot is however, much closer to the origin (offset by only 0.31 unit on the horizontal axis) than the *α* plot, which is quite distant from zero-point origin (with an offset of 1.68 units on the horizontal axis). The reason for this can be seen by closely examining the electrostatic properties of cyclohexane—the solvent used to develop *α* and *β* scales—as they relate to hydrogen-bond acidity and hydrogen-bond basicity. The assumption that *β* = 0 is a reasonable assumption based on the facts that the surface of cyclohexane does not consist of electronegative atoms or electron pairs. However, the same argument does not hold for the *α* scale because the hydrogen atoms on the surface of cyclohexane, consisting of C–H bonds, are polarized slightly towards the carbon atom, therefore the hydrogen atoms possess partial positive charges and they are slightly acidic. As a result, the *α* hydrogen-bond acidity—although expected to be small because the degree of C–H bond polarization in cyclohaxane is also small—should not be zero (if the *α* scale is to have a zero origin). Evidently, the ESP plotted on the van der Waals' surface of cyclohexane shows that the hydrogen atoms have *E*_max_ value of +22.4 kcal mol^–1^, yet the *α* value of cyclohexane is zero. As a result, solvents with *E*_max_ value less than +22.4 kcal mol^–1^ have also been assigned the value of zero even when in reality they are likely to be less acidic than cyclohexane (Fig. 5).

Using the extrapolated –1.68 and –0.31 as the approximated values for *α* and *β* values in the gas phase, respectively, and the value of –1.1 reported elsewhere for π*,^39^eqn (3) predicted –0.31 kcal mol^–1^ as the CH–aryl interaction energy in the gas phase using our molecular balance. Interestingly, this value compares satisfactorily with –0.3 kcal mol^–1^ reported by Datta *et al.*^22^ for the gas-phase energy difference between folded and unfolded conformers of a structurally related molecular balance. An alternative way to verify the predicted value in the gas phase is through DFT single-point energy calculations using optimized structures of the folded and unfolded conformers. The relative energy calculation was carried out with Gaussian 09 software^43^ at B3LYP-D3 level of theory using 6-31G+(d) as the basis set. The DFT-D results show that the folded conformer is more stable than the unfolded conformer in the amount of –0.35 kcal mol^–1^, which is in remarkably good agreement with –0.31 kcal mol^–1^ predicted by eqn (3).

Because one expects London dispersion forces to be rather significant in the gas phase, the estimated interaction energy should be in the region of –1.0 kcal mol^–1^. However, the energy was observed to be –0.35 kcal mol^–1^. This energy difference could be attributed to a considerable cancellation of the attractive dispersion term by an almost equally large repulsive (exchange) steric term, which is likely caused by the bent structure of the molecular balance.

The predicted Δ*G* in the gas phase provides a reference point for gauging the energetic contributions of solvation in the conformational equilibrium of the molecular balance. The results (shown in Table 1) indicated that attractive CH–aryl interactions occurred in all solvents—because of the negative Δ*G* values. However, relative to –0.31 (or –0.35) kcal mol^–1^ predicted in the gas phase, it is apparent that not all the solvents actually enhanced the formation of CH–aryl interactions. In fact, non-polar solvents (cyclohexane, chloroform, methylene chloride, benzene, and carbon tetrachloride) showed a destabilization effect because their Δ*G* values are either equal to or less than the value in the gas phase. The small *α* and *β* values of the non-polar solvents indicate that the CH–aryl interaction in these solvents is dominated by the π* term, *i.e.*, the small diminishing effect can be attributed to competitive London dispersion forces. On the other hand, polar solvents (those with the propensity to act as hydrogen-bond donors) favoured the formation of CH–aryl interactions, which was evidenced by the resulting Δ*G* values for these solvents being greater than the Δ*G* value in the gas phase.

## Conclusions

In addition to providing data on the strength of CH–aryl interactions as a function of solvation, we have also demonstrated that it is possible to use the Kamlet–Taft equation to offer a molecular-level rationale for solvent effects in a *weak* CH–aryl interaction. The magnitude and signs of the correlation coefficients in eqn (3) show that specific solvent effects dominate the influence of solvation on a CH–aryl interaction. Solvents with high *β* value and low *α* value were found to stabilize CH–aryl interactions while solvents with low *β* value and high *α* value disfavour CH–aryl interactions. In addition, the Kamlet–Taft solvation model offers some insight into the energetics of CH–aryl interactions in the gas phase. We find that the predicted Δ*G* in the gas phase lies at the boundary between Δ*G* values for polar solvents and Δ*G* values for non-polar solvents. Consequently, contrary to the notion that noncovalent interactions may be overestimated in the gas phase, we find that Δ*G* values in the gas phase—using the molecular balance approach—are within the context of Δ*G* values in solution. The quantitative partitioning of solvent effects into electron pair acceptor and electron pair donor provides a physical basis for understanding the nature of CH–aryl interactions in solution. Moving forward, we are currently investigating the universality of the Kamlet–Taft equation for modelling solvent effect in other noncovalent interactions.

## Supplementary Material

Supplementary information
