# Supplementary material for: Quantitative model for rationalizing solvent effect in noncovalent CH–Aryl interactions
Source: Chem Sci. 2015 Nov 17;7(2):1401–7. doi: 10.1039/c5sc03550c (PMC5975927; doi:10.1039/c5sc03550c)
Supplement: Supplementary file 1 [file SC-007-C5SC03550C-s001.pdf]

Electronic Supplementary Material (ESI) for Chemical Science.
This journal is © The Royal Society of Chemistry 2015

Supporting Information for

Quantitative Model for Rationalizing Solvent Effect in Noncovalent CH−Aryl Interactions

Bright U. Emenike,*[a] Sara N. Bey[a], Brianna C. Bigelow[a], and Srinivas V.S. Chakravartula[b]

[a] Department of Chemistry & Physics, State University of New York, 223 Store Hill Road, Old Westbury, NY 11568, USA. E-mail: emenikeb@oldwestbury.edu
[b] Department of Chemistry & Biochemistry, Hunter College Graduation Center, City University of New York, 695 Park Avenue New York, NY 10065, USA

Table of Contents

General Experimental 3

Measurement of interaction energies (ΔG) and error analysis 3

Syntheses 4

Synthesis of Balance 1: 4
Synthesis of Balance 2: 4
Synthesis of balance 3: 5
Synthesis of Balance 4: 6

Tables and Figures 7

Table S1. Experimental ΔG values in various solvents and solvent properties. 7
Figure S1. Correlation of interaction energies with solvent polarity scales (a) dipole moment and, (b) dielectric constant. 8
Figure S2. Correlation of ΔGexp with Kamlet-Taft α and β parameters 9
Figure S3. Correlation of ΔGexp with cohesive energy density 9
Table S2. Experimental and predicted ΔG values (kcal/mol) in various solvents using Hunter's Solvation model. 10
Figure S4. Correlation of ΔGexp with Hunter hydrogen bond α and β parameters. 10
Figure S5. Optimized geometries of folded and unfolded conformers of molecular balance 1 at b3lyp/6-31+g(d) level. Image generated with CYLview program 11

Computational method 11

The xyz coordinates for the unfolded conformer 11
The xyz coordinates for the folded conformer 13

Proton & Carbon NMR of Balance 1 15

Proton & Carbon NMR of Balance 2 16

Proton & Carbon NMR of Balance 3 17

Proton & Carbon NMR of Balance 4 18

References 19

General Experimental

All reactions were carried out under an atmosphere of nitrogen in oven-dried glassware with magnetic stirring unless otherwise indicated. Reagents were purchased from commercial sources and used without further purification. Solvents for chemical reaction were dried. Purification of the reaction products was carried out by flash chromatography using silica gel 40-63 µm (230-400 mesh) unless otherwise stated. Reactions were monitored by ^{1}H NMR and/or thin layer chromatography. Visualization was accomplished with UV light, staining with 5% $KMnO_4$ followed by heating or with p-anisaldehyde in EtOH solution. NMR was recorded using a 400 MHz Bruker spectrometer. Chemical shifts were recorded in ppm (□□□using tetramethylsilane (C, H) as the internal reference. Data are reported as (s = singlet, d = doublet, t = triplet, q = quartet, m = multiplet; integration; coupling constant(s) in Hz). Melting points were measured with a Gallenkamp melting point apparatus.

Measurement of interaction energies (ΔG) and error analysis

The *folded/unfolded* ratios were measured by proton NMR spectra at room temperature (~ 25°C). The peak areas corresponding to the *ortho* methyl proton in the *folded* and *unfolded* states were integrated. The CH-π interaction energies were estimated from the equation: ΔG = -RTln$K$ =-RTln[*folded*]/[*unfolded*].

The error in the folding energies was estimated to be 0.03 kcal/mol based on a conservative estimate of the NMR measured *folded*/*unfolded* ratio of ±5%.[1]

Multiple linear regression was performed with *Statplus* program, and the result (with errors included) are as follows:

**Regression Statistics**	
R	0.98
R Square	0.97
Adjusted R Square	0.94
Standard deviation	0.05
# of observations	10

	**Coefficients**	**Standard Error**
Intercept	-0.24	0.04
α	0.23	0.05
β	-0.68	0.1
π*	-0.1	0.09
δ	0.09	0.05

Syntheses

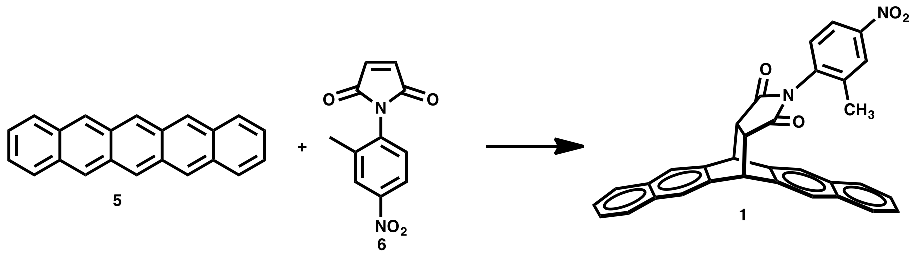

Synthesis of Balance 1:

Pentacene (1 mmol, 278 mg), prepared according to literature procedure,[2] and 1 mmol of known imide[3] (**6**) were mixed in a reaction tube containing 5ml toluene. The reaction tube was sealed under nitrogen atmosphere and was placed in an oil bath at 100°C. After 5 h, the dark-blue reaction solution became clear, leaving a light yellow solution. The reaction tube was cooled to room temperature and a crystalline white product (**1**) was collected by filtration. Further purification of **1** was achieved by a flash column chromatography using ethyl acetate-hexane mixture. Yield > 99% and m.pt > 250°C. ^{1}H NMR (400 MHz, DMSO-d_6) δ 0.54 (s, 2H), 2.16 (s, 1H), 3.66 (s, 2H), 5.13 (s, 2H), 7.37 (d, 1H, J = 8.8 Hz), 7.47-7.52 (m, 4H), 7.87-7.93 (m, 7H), 8.04-8.08 (m, 3H). ^{13}C NMR (400 MHz, DMSO-d_6) 15.9, 17.9, 45.2, 45.7, 47.3, 47.4, 121.6, 121.8, 123.0, 123.2, 123.9, 124.3, 125.6, 126.4, 126.5, 126.6, 127.7, 127.8, 128.4, 129.2, 132.5, 132.6, 132.9, 135.6, 135.8, 136.4, 137.7, 137.8, 138.2, 138.4, 147.8, 147.9, 175.3, 175.4 HRMS calcd for $C_{33}H_{22}N_2O_4$ $[M+H]^+$ 511.1580 found 511.1640

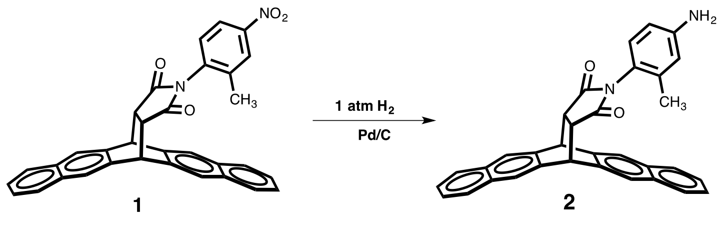

Synthesis of Balance 2:

Hydrogenation was carried out following a literature procedure.[4] Balance **1** (1mmol) was dissolved in 5 ml of ethyl acetate, and mg of Pd/C (10%) was added. The mixture was stirred at room temperature for 24 h under a hydrogen atmosphere (using balloon). The solution was filtered through a pad of celite and concentrated to dryness. The crude

product was purified by flash chromatography (50% solution of hexanes and ethyl acetate). Yield = 92% and m. pt > 250°C. ^{1}H NMR (400 MHz, DMSO-d_6) δ 0.13 (s, 2H), 1.74 (s, 1H), 3.48 (s, 2H), 5.01-5.05 (m, 4H), 5.98 (d, 1H, *J* = 2.4 Hz), 6.21 (m, 1H), 6.49 (d, 1H, *J* = 8.4 Hz), 7.39 (m, 4H), 7.79 (m, 6H), 7.99 (m, 2H). ^{13}C NMR (400 MHz, DMSO-d_6) δ 15.6, 17.8, 44.8, 45.3, 46.7, 46.8, 112.2, 115.3, 120.1, 123.0, 123.2, 123.2, 123.8, 124.1, 125.8, 126.5, 127.9, 128.1, 128.7, 128.9, 129.4, 132.5, 132.8, 135.9, 137.0, 137.3, 139.3, 139.7, 149.2, 177.0 HRMS calcd for $C_{33}H_{24}N_2O_2$ $[M+H]^+$ 481.1838 found 481.1908

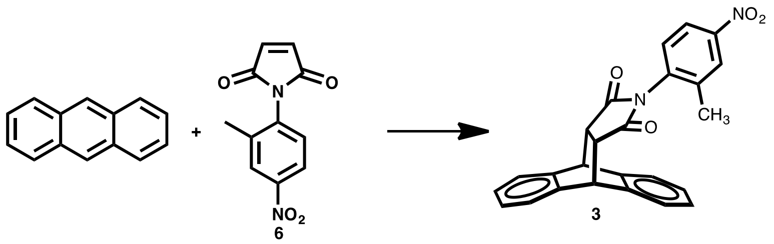

Balance **3** is a known compound.[3]

Synthesis of balance 3:

Anthracene (1 mmol) and 1 mmol of *N*-(2'-methyl-4-nitrophenyl)maleimide (**6**) was dissolved in 5 ml xylene. The mixture was refluxed overnight under nitrogen atmosphere. The reaction mixture was concentrated using rotary evaporator and the crude product was purified by flash chromatography to afford balance **3** in 88% yield. The NMR data were consistent with those reported elsewhere.[3] ^{1}H NMR (400 MHz, $CDCl_3$) δ 1.07 (s, 1H), 2.08 (s, 2H), 3.44 (s, 2H), 4.92 (d, 2H, *J* = 7.2 Hz), 5.53 (d, 1H, *J* = 7.6 Hz), 6.93-7.28 (m, 11 H). ^{13}C NMR (400 MHz, DMSO-d_6) 16.3, 17.7, 44.9, 45.4, 47.6, 122.1, 124.8, 124.9, 125.4, 125.6, 126.8, 126.9, 127.3, 127.4, 130.3, 137.8, 138.5, 139.8, 140.3, 141.9, 142.6, 147.9, 175.9

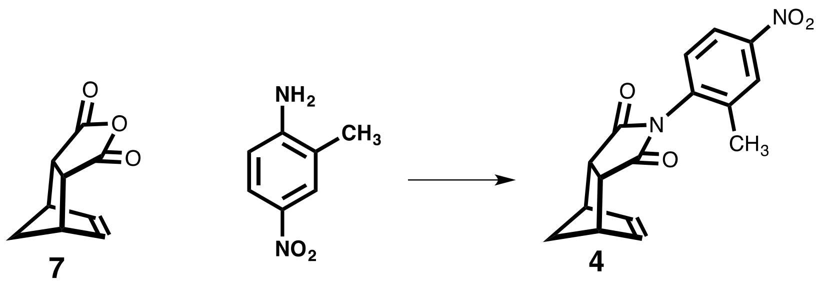

Synthesis of Balance 4:

Procedure: 10 mmol of 2-methyl-4-nitroaniline was added under stirring to a solution containing 10 mmol of known anhydride (**7**) and 0.5 ml triethylamine in 10 ml of DMF. The mixture was heated at 120°C for 6h in a sealed tube after which the reaction was cooled to room. The mixture was poured in 50 ml of 10% HCl solution and the aqueous solution was extracted three times with 15 ml portions of diethyl ether. The combined organic extract was successively washed with saturated brine and water. The organic solvent, after being dried with $MgSO_4$, was removed with rotary evaporator. Purification of product was achieved by flash chromatography to afford **3** in 76% yield. ^{1}H NMR (400 MHz, $CDCl_3$) δ 1.65 (t, 1H), 1.84 (t, 1H), 2.21 (d, 3H, J = 11.6 Hz), 3.52 (s, 4H), 6.32 (s, 2H), 7.04-7.17 (dd, 1H, J = 8.4 Hz), 8.07-8.17 (m, 2H). ^{13}C NMR (400 MHz, DMSO-d_6) 17.7, 18.8, 45.1, 45.4, 46.2, 47.2, 52.3, 52.8, 122.1, 122.1, 125.8, 125.9, 130.1, 135.1, 135.7, 137.7, 138.3, 138.6, 138.8, 147.9, 176.5, 176.7. HRMS calcd for $C_{16}H_{14}N_2O_4$ $[M+H]^+$ 299.0954 found 299.1012

Tables and Figures

Table S1. Experimental ΔG values in various solvents and solvent properties.

solvent	ε	μ	$E_T(30)$	α	β	π^*	*ced*	ΔG (exp) kcal/mol Balance 4	ΔG (exp) kcal/mol Balance 1
cyclohexane	2.0	0.0	30.9	0.00	0.00	0.00	66.9	0.02	-0.22
chloroform	4.8	1.2	39.1	0.20	0.10	0.58	85.4	0.00	-0.26
CH_2Cl_2	8.9	1.6	40.7	0.13	0.10	0.82	93.7	0.02	-0.26
CCl_4	2.2	0.0	32.4	0.00	0.10	0.28	73.6	-0.02	-0.31
benzene	2.3	0.0	34.3	0.00	0.10	0.59	83.7	-0.02	-0.34
pyridine	12.3	2.4	40.5	0.00	0.64	0.87	112.4	-0.12	-0.62
DMSO	47.0	3.9	45.1	0.00	0.76	1.00	169	-0.17	-0.90
1,4-dioxane	2.2	0.5	36.0	0.00	0.37	0.55	94.7	-0.07	-0.53
methanol	32.6	1.7	55.4	0.98	0.66	0.60	209	-0.07	-0.50
acetic acid	6.2	1.7	51.7	1.12	0.45	0.64	109.2	-0.02	-0.37
acetone	21.0	2.9	42.2	0.08	0.43	0.71	94.3	-0.12	-0.59
acetonitrile	36.6	3.5	45.6	0.19	0.40	0.75	139	-0.10	-0.59
THF	7.5	1.8	37.4	0.00	0.55	0.58	86.9	-0.12	-0.68
nitromethane	35.9	3.5	46.3	0.22	0.06	0.85	158.8	-0.02	-0.34

Symbols: ε = dielectric constant; μ = dipole moment in Debye unit; $E_T(30)$ = Reichardt's solvent polarity in kcal/mol; α, β, and π^* are Kamlet-Taft's hydrogen-bond acidity, hydrogen-bond basicity, and polarity/dipolarity of solvents, respectively. Cohesive energy density = ced (cal cm^{-3}), see reference[5].

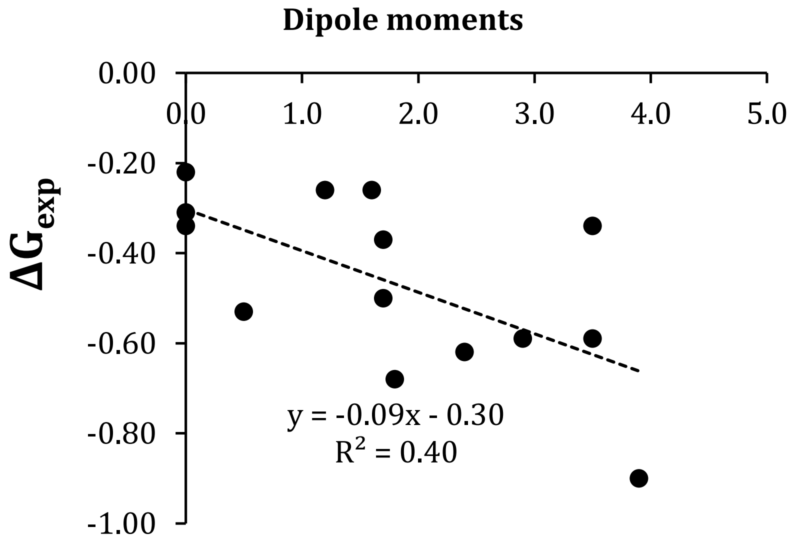

(a)

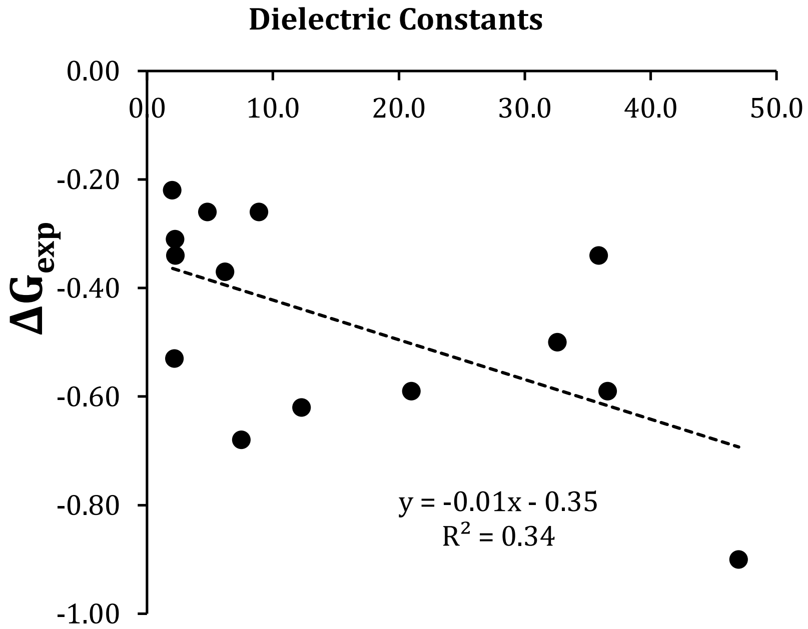

(b)

Figure S1. Correlation of interaction energies with solvent polarity scales (a) dipole moment and, (b) dielectric constant.

$$\Delta G_{pred} = -2.26 + 0.22\alpha - 0.75\beta$$

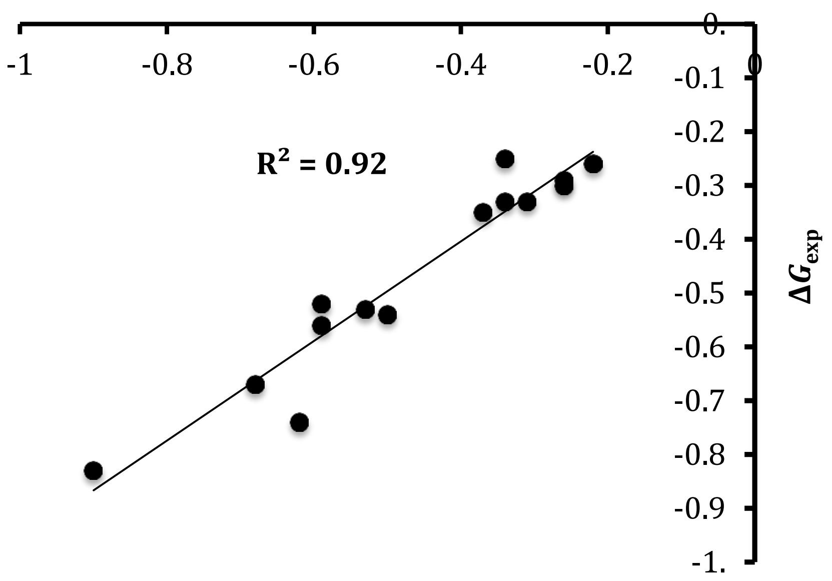

Figure S2. Correlation of ΔGexp with Kamlet-Taft α and β parameters

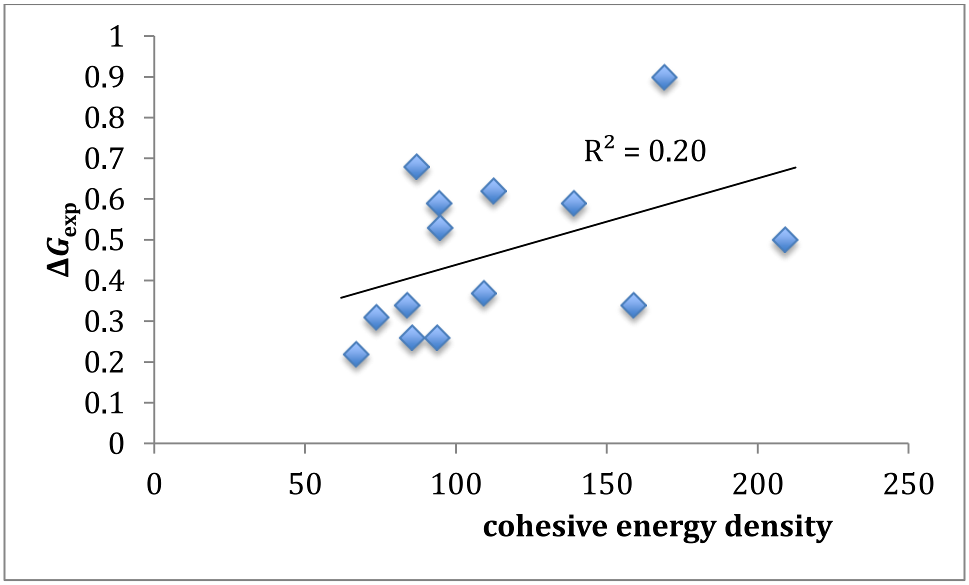

Figure S3. Correlation of ΔGexp with cohesive energy density

Table S2. Experimental and predicted ΔG values (kcal/mol) in various solvents using Hunter's Solvation model.

Solvent	α_s	β_s	Experimental ΔG (kcal/mol)	Predicted ΔG (kcal/mol)
Chloroform	2.1	0.9	-0.26	-0.22
Acetone	1.5	5.7	-0.59	-0.58
Acetonitrile	1.7	5.1	-0.59	-0.53
Benzene	1.1	2.1	-0.34	-0.37
Cyclohexane	1.0	0.6	-0.22	-0.27
THF	0.9	5.9	-0.68	-0.64
DCM	1.9	1.1	-0.26	-0.25
Methanol	2.7	5.3	-0.50	-0.48
DMSO	0.8	8.9	-0.90	-0.85
CCl_4	1.4	0.6	-0.31	-0.25
Acetic acid	3.7	4.9	-0.37	-0.39
1,4-dioxane	0.9	4.7	-0.53	-0.56
Nitromethane	1.8	3.7	-0.34	-0.43
Pyridine	1.2	7.2	-0.62	-0.71

The predicted ΔG values are derived from Hunter solvent hydrogen bond parameters, α_s and β_s.[6, 7]

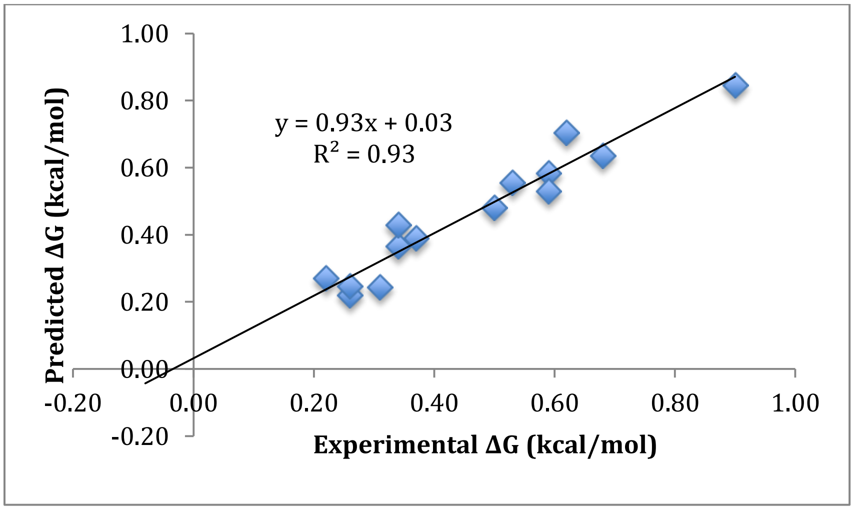

Figure S4. Correlation of ΔGexp with Hunter hydrogen bond α and β parameters

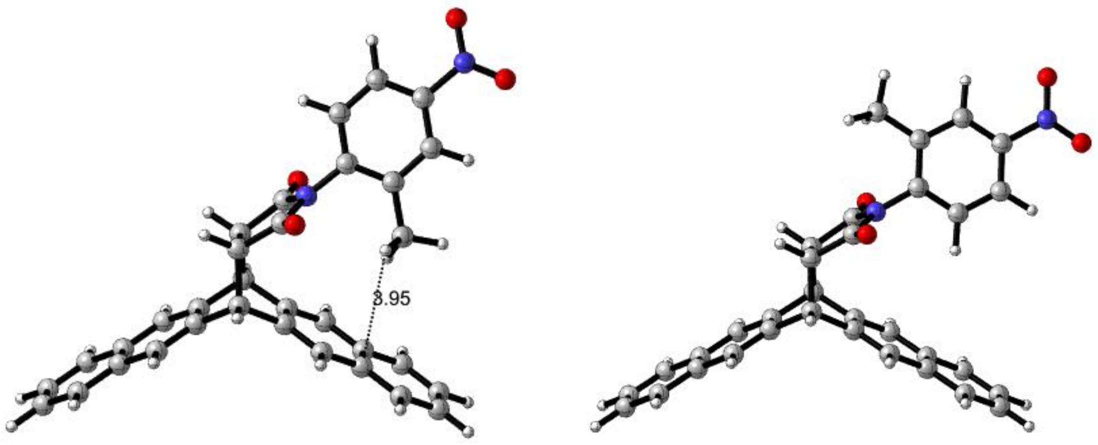

Figure S5. Optimized geometries of folded and unfolded conformers of molecular balance 1 at b3lyp/6-31+g(d) level. Image generated with CYLview program

Computational method

Conformational search for the "folded" and "unfolded" conformers began by performing Monte Carlo molecular mechanics calculation with MMFF force field (Spartan '10). The "conformational distribution" option was utilized.

Two low-energy conformers were found and the structures were then re-optimized at B3LYP/6–31+G(d) using the Gaussian 09 program suite. In order to ensure the optimized structures are stable conformers and not transition-state conformers, frequency calculations were performed using the same level of theory applied in the optimization calculations, which resulted imaginary frequencies of zero for both conformers.

Single-point energy calculations of the optimized conformers were performed using a dispersion-corrected B3LYP-D3 level of theory with 6-31+G(d) basis set.

Total Energies

Level	Folded (a.u)	Unfolded (a.u)	Difference (kcal/mol)
B3LYPD3/6-31G+(d)	-1681.277332	-1681.276774	-0.35

The xyz coordinates for the unfolded conformer

C	1.70806	-0.41419	-1.24679
H	1.68817	-0.48985	-2.33632
C	1.75367	-0.20529	1.35281
H	1.77193	-0.10719	2.4405
C	1.03787	-1.53125	0.93511
H	1.52085	-2.37702	1.43153
C	1.02914	-1.66567	-0.60208
H	1.52922	-2.5711	-0.9552
C	-0.4378	-1.71531	-0.98892

C	-0.42828	-1.48536	1.33387
O	-0.87587	-1.3757	2.45001
O	-0.89262	-1.83062	-2.10287
N	-1.20042	-1.56116	0.16995
C	-2.60575	-1.31477	0.14601
C	-3.03431	-0.00918	0.3695
C	-3.49853	-2.35987	-0.11258
C	-4.38785	0.29103	0.33718
C	-4.85732	-2.04959	-0.14741
C	-5.27513	-0.74404	0.07504
H	-4.74941	1.29828	0.5029
H	-5.59486	-2.81889	-0.34554
N	-6.7159	-0.44745	0.03172
O	-7.06364	0.70695	0.23159
O	-7.48182	-1.37097	-0.20157
H	-1.21656	3.94382	2.26667
C	-1.2572	3.84566	1.1845
C	-2.03913	4.69238	0.4383
C	-0.49423	2.8235	0.56082
C	-2.09254	4.55891	-0.96962
H	-2.62181	5.46764	0.9274
C	-0.5451	2.69156	-0.85668
C	0.28584	1.90359	1.31705
C	-1.36104	3.58342	-1.60068
H	-2.71844	5.23034	-1.55007
C	0.1969	1.65268	-1.4868
C	0.98348	0.91339	0.6839
H	0.29392	1.97862	2.40231
H	-1.40485	3.47515	-2.68158
C	0.94693	0.79172	-0.73541
H	0.13773	1.53626	-2.56669
C	3.1098	-0.36429	-0.68181
C	4.27359	-0.42646	-1.39474
C	3.13503	-0.25522	0.73918
C	5.52851	-0.37483	-0.72385
H	4.25494	-0.51145	-2.47963
C	4.32385	-0.20709	1.41094
C	5.55406	-0.26251	0.69568
C	6.75777	-0.43024	-1.4307
H	4.34475	-0.11933	2.49559
C	6.80811	-0.20672	1.35753
C	7.9563	-0.37493	-0.76315
H	6.73544	-0.51725	-2.51443
C	7.98181	-0.26106	0.64692
H	6.82559	-0.11731	2.44116
H	8.89051	-0.41764	-1.31556

H	8.93515	-0.21571	1.16531
C	-3.01293	-3.76301	-0.35388
H	-2.50905	-3.8332	-1.32382
H	-2.30035	-4.07142	0.41826
H	-3.84633	-4.47011	-0.34932
H	-2.30217	0.76854	0.56226

The xyz coordinates for the folded conformer

C	-1.62901	-0.42105	1.28296
H	-1.61722	-0.46413	2.37444
C	-1.6608	-0.28829	-1.32403
H	-1.67607	-0.22054	-2.41415
C	-0.80093	-1.50233	-0.86016
H	-1.18832	-2.41126	-1.32985
C	-0.8014	-1.59509	0.67968
H	-1.21702	-2.53996	1.04163
C	0.6607	-1.53808	1.08307
C	0.66267	-1.36306	-1.24614
O	1.11253	-1.22895	-2.35947
O	1.10921	-1.58149	2.20364
N	1.43009	-1.42187	-0.07925
C	2.85688	-1.36589	-0.06145
C	3.50771	-0.14937	-0.30034
C	3.55698	-2.53544	0.21871
C	4.90136	-0.14832	-0.265
C	4.9439	-2.52511	0.25961
C	5.58932	-1.32207	0.01054
H	5.45528	0.76626	-0.44332
H	5.51313	-3.4202	0.47698
N	7.06035	-1.28642	0.04768
O	7.61036	-0.21755	-0.17208
O	7.6468	-2.32932	0.29656
H	0.46259	4.33891	-2.30412
C	0.49312	4.28011	-1.21869
C	1.04573	5.29937	-0.48321
C	-0.04319	3.1318	-0.58047
C	1.08609	5.21833	0.92895
H	1.45616	6.17193	-0.98306
C	-0.00381	3.05063	0.84114
C	-0.59064	2.04852	-1.32328
C	0.57167	4.12096	1.5741
H	1.52734	6.0294	1.50087
C	-0.52083	1.89164	1.48698

C	-1.07672	0.94807	-0.67596
H	-0.60278	2.10082	-2.41007
H	0.60492	4.05439	2.6589
C	-1.04819	0.87054	0.7468
H	-0.48222	1.82486	2.57223
C	-3.01898	-0.57136	0.69937
C	-4.17505	-0.771	1.3987
C	-3.03571	-0.50356	-0.72523
C	-5.4147	-0.90872	0.71088
H	-4.16293	-0.82481	2.4857
C	-4.20898	-0.63392	-1.41238
C	-5.43212	-0.83799	-0.71142
C	-6.63601	-1.11457	1.40323
H	-4.22381	-0.57914	-2.49931
C	-6.67055	-0.97414	-1.39045
C	-7.81954	-1.24401	0.71914
H	-6.62003	-1.16903	2.48921
C	-7.83709	-1.17241	-0.69369
H	-6.68204	-0.91731	-2.47638
H	-8.74806	-1.40123	1.26024
H	-8.7788	-1.27551	-1.22492
H	3.01042	-3.45304	0.40969
C	2.73466	1.10958	-0.57081
H	1.96413	1.26258	0.1907
H	3.39031	1.98387	-0.56907
H	2.23165	1.0569	-1.5416

Proton & Carbon NMR of Balance 1

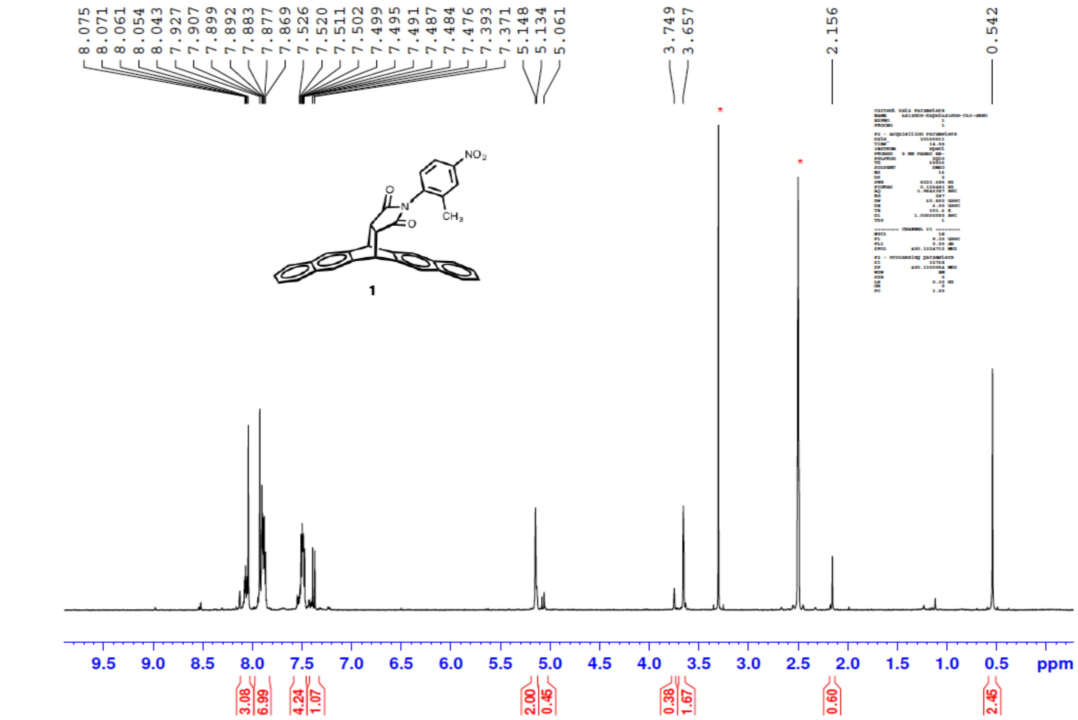

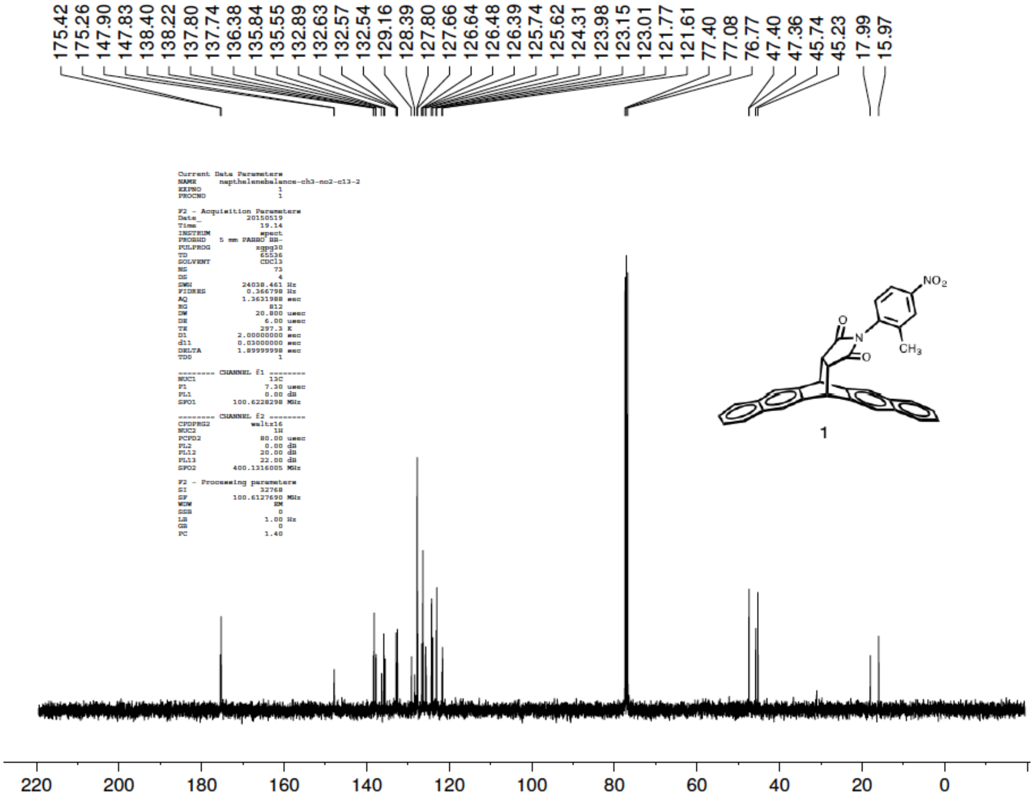

Proton & Carbon NMR of Balance 2

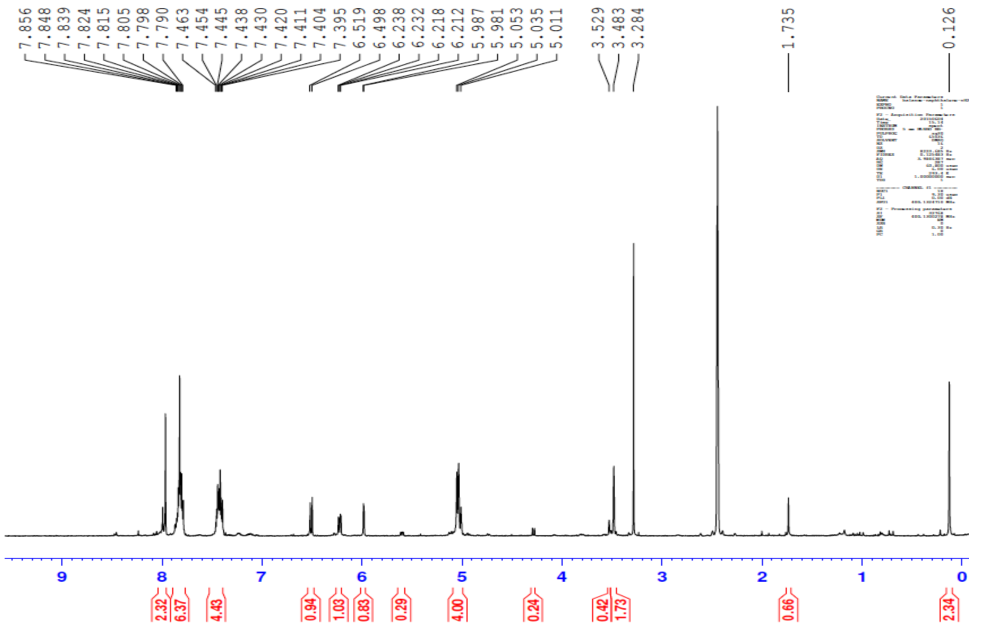

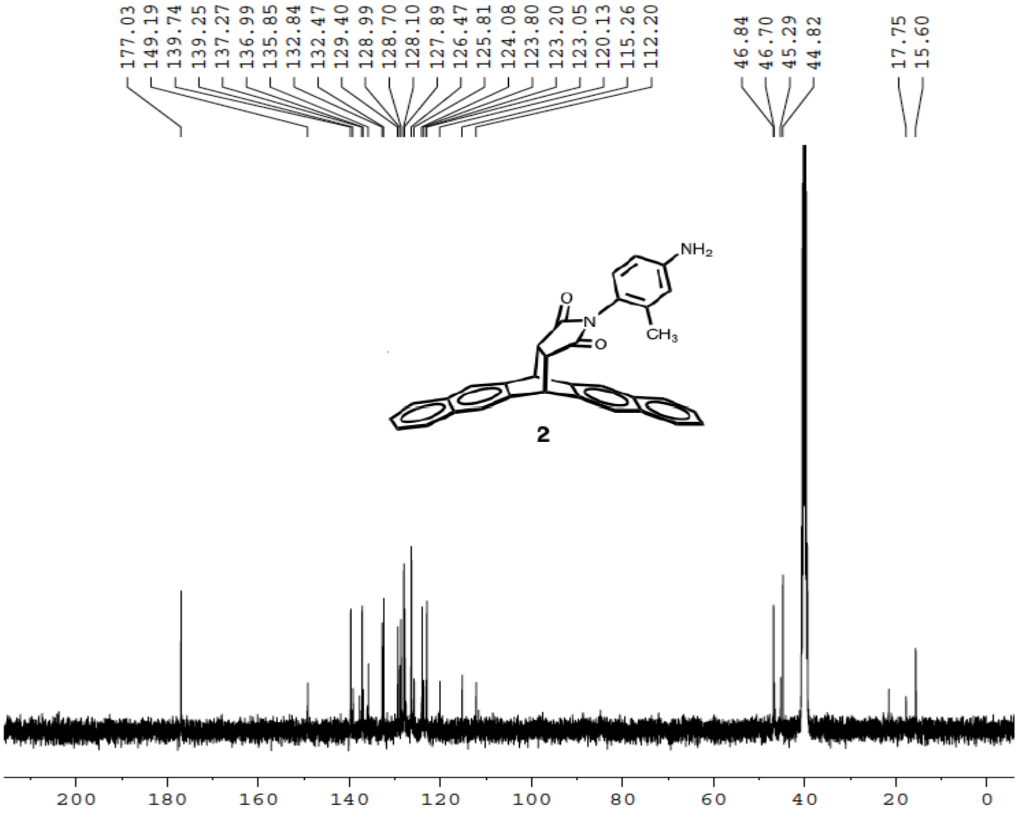

Proton & Carbon NMR of Balance 3

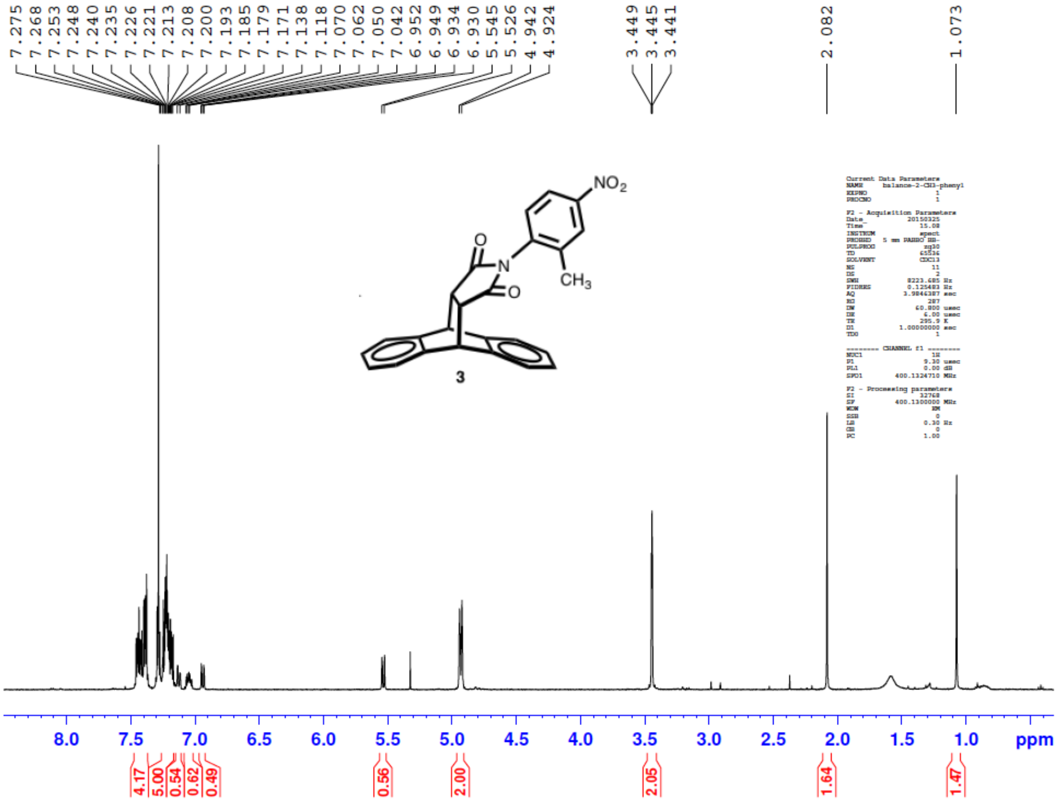

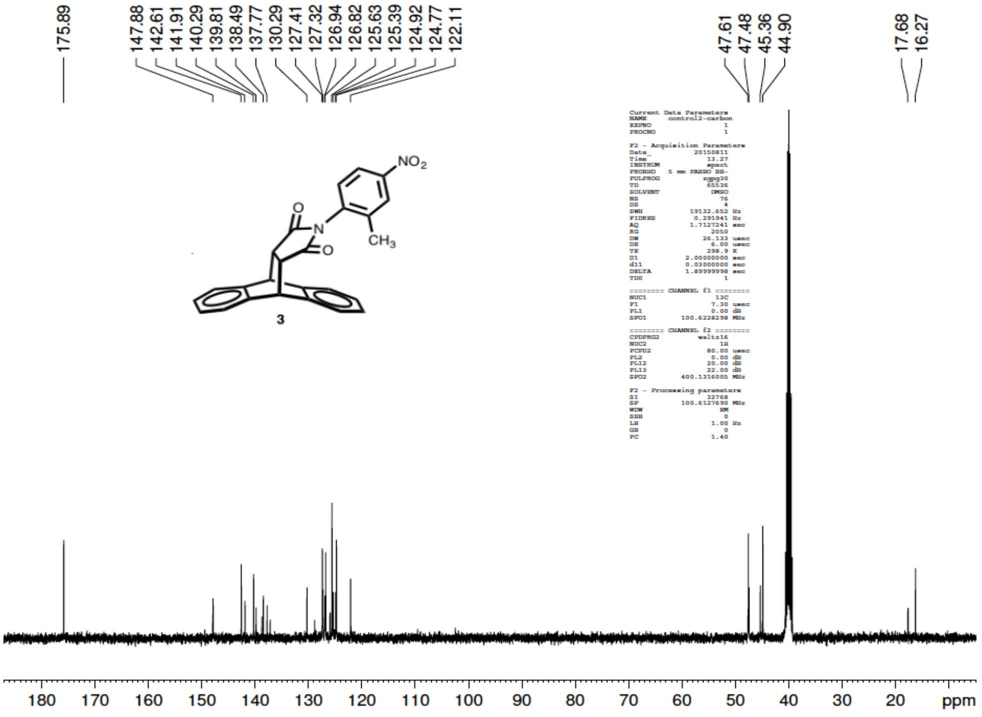

Proton & Carbon NMR of Balance 4

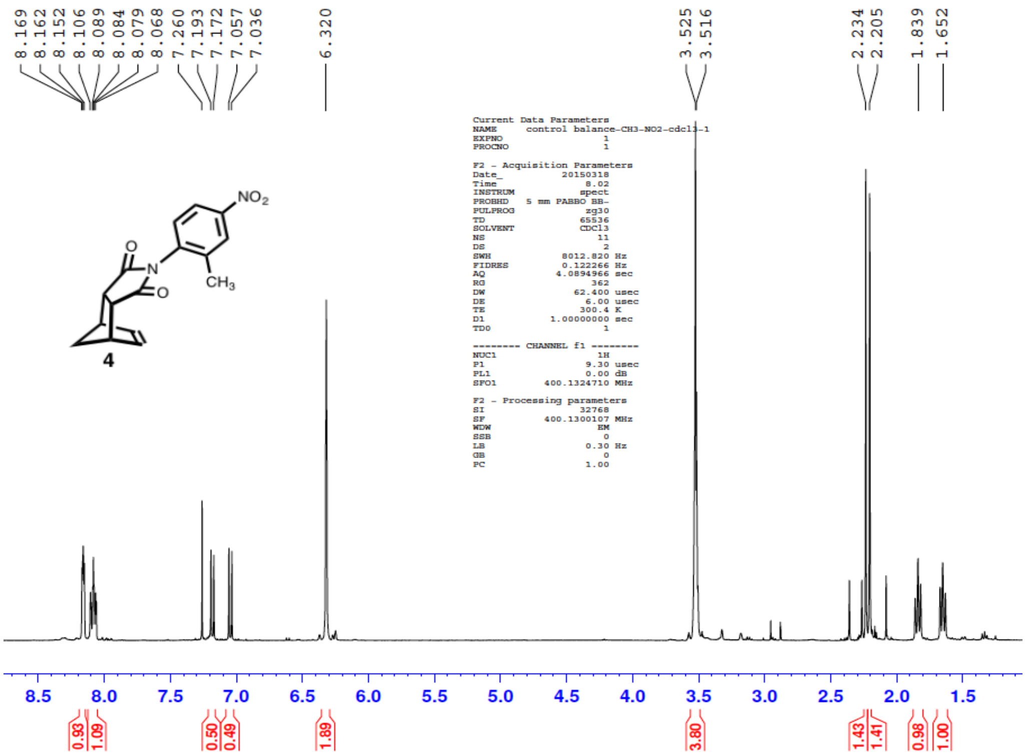

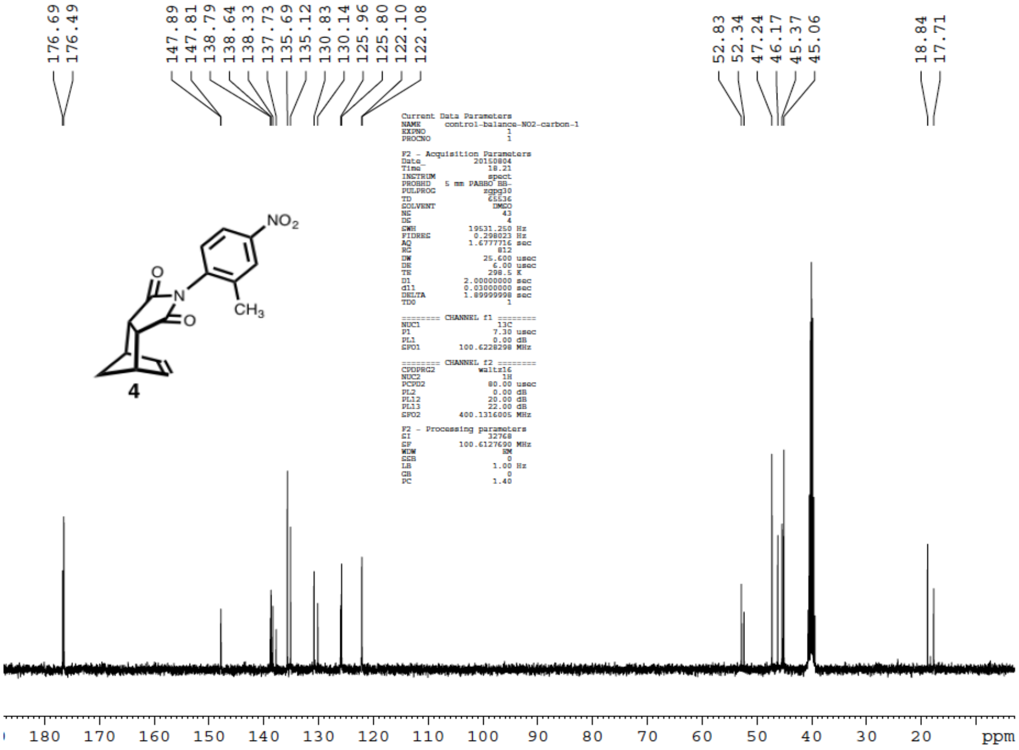

References

1. M. Bauer, A. Bertario, G. Boccardi, X. Fontaine, R. Rao and D. Verrier, *J. Pharm. Biomed. Anal.*, 1998, **17**, 419-425.
2. C. Pramanik and G. P. Miller, *Molecules*, 2012, **17**, 4625-4633.
3. K. Kishikawa, K. Yoshizaki, S. Kohmoto, M. Yamamoto, K. Yamaguchi and K. Yamada, *Journal of the Chemical Society-Perkin Transactions 1*, 1997, 1233-1239.
4. M. Lanier, D. Schade, E. Willems, M. Tsuda, S. Spiering, J. Kalisiak, M. Mercola and J. R. Cashman, *J. Med. Chem.*, 2012, **55**, 697-708.
5. L. Yang, C. Adam and S. L. Cockroft, *J. Am. Chem. Soc.*, 2015, **137**, 10084-10087.
6. C. A. Hunter, *Angewandte Chemie-International Edition*, 2004, **43**, 5310-5324.
7. J. L. Cook, C. A. Hunter, C. M. R. Low, A. Perez-Velasco and J. G. Vinter, *Angewandte Chemie-International Edition*, 2007, **46**, 3706-3709.
